# Applied Physiology in Practice

**Published:** 1912-06-29

**Authors:** 


### THE MAKING OF THE MODERN PRACTITIONER.

### Applied Physiology in Practice.

Faced with the extraordinary strides which scientific research of all sorts has been making during recent years, the modern practitioner is somewhat in the position of having to put new wine into old bottles. We all know the traditional result of this manœuvre; nevertheless it can be carried out not only without disaster but with complete success. The effort and labour required are, it is true, considerable; but they are well worth the exertion. It is, of course, not every man in medical practice who has the laudable itch for keeping himself abreast of the times. Not long ago a middle-aged—and very shrewd—general practitioner was heard denouncing one of his younger neighbours as being " too scientific for me "; and this attitude of mind is not uncommon. Yet there are many, especially of those who have left the hospitals within the last fifteen years, who strain to keep in touch with all that is worth knowing of modern progress.

Nor is it only the general practitioner who finds it difficult to discriminate between the wheat and the chaff of contemporary novelties. Surgery and physiology are becoming both such vast subjects that it is very difficult for a devotee of either to keep track of the advances made in the other. This is lamentable, for the two sciences are complementary at so many points of mutual contact that a knowledge of both is and must be of paramount value to the student of either.

Fortunately, an able writer * has lately set himself to bridge some of the gaps we have alluded to, and to sum up in succinct form the most important of the facts established by physiologists in recent years beyond likelihood of subversion. With great pleasure we note that a second edition of this book has been required within a few months of the publication of the first one (*vide* The Hospital, April 6, 1912, p. 22); and the reason for our pleasure is that the rapid sale of this book is evidence that the profession in the bulk is willing to be taught when it can find a capable teacher. Iconoclastic as Dr. Short is, his chapters are founded on accurate statement and logical reasoning; and his easy style and lack of affectation make them pleasant reading—even, we think, for those whose pet dogmata are the most mercilessly criticised.

In this second edition several new discoveries are included, made since the first edition went to press. Thus the first chapter, which is entirely new matter, deals with Sir William Macewen's researches into the growth of bone; the revolutionary hypotheses propounded so ably by this surgeon were epitomised in The Hospital on April 6. No doubt Dr. Short would in any case have made himself acquainted with the Scottish surgeon's researches; but we should like to think that the coincidence of the two subjects (of applied physiology and of bony growth) being discussed in the same issue of this journal has had something to do with directing Dr. Short's attention to the matter.

In the chapter on surgical shock a full account of the quite conflicting and apparently irreconcilable hypotheses of Crile and Yandell Henderson is given. Like most other clinicians, the author is inclined to suspend judgment until the exact bearings of all the collected facts can be gauged; but it is evident that Henderson's fascinating theses have attracted him strongly, as they do all who study them. The practitioner may ask what is the practical upshot of the " shock " controversy; and at present, it must be admitted, that it is not easy to say. For what one school endorses, the other contemptuously rejects. Crile and his followers discredit strychnine utterly; Henderson and his friend give it liberally in certain cases; and so with pituitrin, suprarenal extract, and other measures for treating shock. It is here that the opportunity of the surgeon and of the general practitioner arises. If the latter can be sure, from clinical observations, that any one drug (say, pituitrin) really does ameliorate the symptoms of surgical shock, then any hypothesis such as Henderson's, which treats that substance as dangerous and useless, cannot as it stands be accepted. The difficulty is to be sure of one's ground as to any given drug; for the cardinal principles of warmth, recumbency, transfusion, and so forth, are of course adopted contemporaneously, and hence the undiluted effect of any drug can never in clinical work be determined beyond the possibility of doubt. Still, the point remains that the hypotheses of physiologists cannot be allowed to overrule the clinical experience of the practitioner. The physiologist may suggest forms of treatment, based upon experimental and theoretical reasonings; but the last word is with the clinician, who alone can decide whether the suggestions are of practical value.

Of the many other instructive topics dealt with, we have space to mention briefly but two. One is the therapeutic uses of calcium salts, on which the author barely touches. He says, quite truly, that calcium given internally " may act like a charm " for chilblains. Can he explain how it is that it also " may " have no remedial effect whatsoever? and can he afford any criterion by which clinicians can distinguish between those who will be benefited and those who will not? Lastly, we would draw attention to his remarks on the use of sedative drugs—laudanum, belladonna, and the like—to the unbroken skin. The old belief in them dies very hard, but even the sceptical gentleman above mentioned who objected to " scientific " newcomers would probably reconsider some of his prejudices if he could be got to study Dr. Short's remarks on this topic. It is, we repeat, a good sign that the " New Physiology " has attracted such a wide circle of readers.

---

* *The New Physiology in Surgical and General Practice.* By A. Rendle Short, M.D., F.R.C.S. Second Edition. (Bristol : J. Wright and Sons. 1912. Price 4s. 6d.)

